# Pyrrolidine dithiocarbamate ameliorates endothelial dysfunction in thoracic aorta of diabetic rats by preserving vascular DDAH activity

**DOI:** 10.1371/journal.pone.0179908

**Published:** 2017-07-17

**Authors:** Chang-Wu Lu, Yuan Lin, Yan-Ping Lei, Lan Wang, Zhi-Min He, Yan Xiong

**Affiliations:** 1 Department of Pharmacology, School of Pharmaceutical Sciences, Central South University, Changsha, Hunan, P. R. China; 2 Guangzhou Institute of Snake Venom Research, Guangzhou Medical University, Guangzhou, Guangdong, P. R. China; 3 Cancer Hospital and Cancer Research Institute of Guangzhou Medical University, Guangzhou, Guangdong, P. R. China; Universita degli Studi di Padova, ITALY

## Abstract

**Objective:**

Endothelial dysfunction plays a pivotal role in the development of diabetic cardiovascular complications. Accumulation of endogenous nitric oxide synthase (NOS) inhibitor asymmetric dimethylarginine (ADMA) and inhibition of dimethylarginine dimethylaminohydrolase (DDAH) activity have been involved in diabetic endothelial dysfunction. This study was to investigate the effect of pyrrolidine dithiocarbamate (PDTC) on impairment of endothelium-dependent vasodilatation in diabetic rats and its potential mechanism.

**Methods:**

Diabetic rats were induced by a single intraperitoneal injection of streptozotocin (60mg/kg), and PDTC (10mg/kg) was given in drinking water for 8 weeks. Blood glucose and serum ADMA concentrations were measured in experimental rats. Recombinant adenovirus encoding human DDAH2 gene were constructed and *ex vivo* transferred to isolated rat aortas. The maximal relaxation (E_max_) and half maximal effective concentration (EC_50_) of aortic rings response to accumulative concentrations of acetylcholine and vascular DDAH activity were examined before and after gene transfection.

**Results:**

Diabetic rats displayed significant elevations of blood glucose and serum ADMA levels compared to control group (*P*<0.01). Vascular DDAH activity and endothelium-dependent relaxation of aortas were inhibited, as expressed by the decreased E_max_ and increased EC_50_ in diabetic rats compared to control rats (*P*<0.01). Treatment with PDTC not only decreased blood glucose and serum ADMA concentration (*P*<0.01) but also restored vascular DDAH activity and endothelium-dependent relaxation, evidenced by the higher E_max_ and lower EC_50_ in PDTC-treated diabetic rats compared to untreated diabetic rats (*P*<0.01). Similar restoration of E_max_, EC_50_ and DDAH activity were observed in diabetic aortas after DDAH2-gene transfection.

**Conclusions:**

These results indicate that PDTC could ameliorate impairment of endothelium-dependent relaxation in diabetic rats. The underlying mechanisms might be related to preservation of vascular DDAH activity and consequent reduction of endogenous ADMA in endothelium via its antioxidant action. This study highlights the therapeutic potential of PDTC in impaired vasodilation and provides a new strategy for treatment of diabetic cardiovascular complications.

## 1. Introduction

Diabetes mellitus is the most prevalent and serious metabolic disease. The leading causes of morbidity and mortality among diabetic population are cardiovascular complications. Endothelial dysfunction has been recognized as the early symbol of diabetic cardiovascular complications, and it is characterized by the impairment of endothelium-dependent relaxation mediated by nitric oxide (NO) [1–3]. Endothelial-derived NO is synthesized from its precursor L-arginine catalyzed by nitric oxide synthase (NOS) in endothelial cells [4]. Asymmetric dimethylarginine (ADMA), a homologue of L-arginine, has been identified as the endogenous NOS inhibitor and an independent risk factor for endothelial dysfunction [5]. The major metabolic pathway of ADMA is to be hydrolyzed by the enzyme dimethylarginine dimethylaminohydrolase (DDAH). There are two isoforms of DDAH, DDAH1 and DDAH2. The former is typically located in neuronal tissues of expressing neuronal NOS (nNOS), and the latter predominates in cardiovascular tissues of expressing endothelial NOS (eNOS) [6]. Therefore, DDAH2 is the key regulator of endogenous ADMA level and NO production in vascular endothelium.

Previous study from the authors’ laboratory demonstrated for the first time that serum level of endogenous NOS inhibitor ADMA was significantly elevated in diabetic rats and associated with the impairment of endothelium-dependent relaxation of thoracic aorta [7]. Later studies showed that elevated endogenous ADMA concentration not only associated with macrovascular complications in diabetic patients, but also related to metabolic control in diabetic rats [8–9]. Lin et al proved that accumulation of endogenous ADMA in endothelium was related to the inhibition of vascular DDAH activity, instead of the suppression of vascular DDAH expression in diabetic rats [10]. Inhibiting DDAH activity of rat aortas via incubation with glycosylated bovine serum albumin (GBSA) *in vitro* or intravenous injection of GBSA to normal rats could lead to endothelial dysfunction [11–12]. Overexpression of DDAH2 could increase vascular DDAH activity and ameliorate hyperglycemia-induced endothelial dysfunction [13]. Accordingly, it is of significance to look for the effective medicine of preserving vascular DDAH activity for the prevention and treatment of endothelial dysfunction and diabetic cardiovascular complications.

Pyrrolidine dithiocarbamate (PDTC) is recognized as the inhibitor of nuclear factor-κB (NF-κB) in a variety of cells. NF-κB is a homo- or hetero-dimeric formed by two members of the Rel protein family such as p65 and p50. Under nonstimulated state, NF-κB resides in the cytoplasm in an inactive form by combination with its inhibitor IκB. In an inflammatory response, pathogenic stimuli cause activation of IκB kinase (IKK) and subsequent phosphorylation of IκB (P-IκB), leading to release of IκB or degradation and then allowing NF-κB to enter the nucleus, in which NF-κB binds to DNA specific sequences and regulates the transcription of various target genes of promoting inflammatory production such as tumor necrosis factor-alpha (TNF-α)[14]. PDTC has been demonstrated to prevent adhesion molecules expressed in endothelial cells [15] and to inhibit inflammatory cytokines expression in esophageal adenocarcinoma cells [16]. PDTC has also been proven to possess the antioxidative or free radical-scavenging properties as a thiol-containing compound [17]. It has been reported that PDTC protected DDAH activity from the inhibition induced by oxidative low density lipoprotein or by homocysteine in cultured endothelial cells [18–19]. However, whether PDTC preserves diabetic vascular DDAH activity and improves endothelial dysfunction *in vivo* of diabetic rats remains unknown. Therefore, the present study was to investigate the effect of PDTC on impairment of endothelium-dependent relaxation and vascular DDAH activity in streptozotocin (STZ)-induced diabetic rats, and to further compare it with the effect of *ex vivo* DDAH transfection, so as to explore the underlying mechanism by which PDTC ameliorates diabetes-induced impairment of endothelium-dependent vasodilation.

## 2. Materials and methods

### 2.1 Reagents

Phenylephrine, acetylcholine (ACh), sodium nitroprusside (SNP), PDTC, antipyrine, diacetyl monoxime, STZ and ADMA were purchased from Sigma Company (St Louis, MO, USA). Recombinant adenovirus Ad5CMV-hDDAH2 encoding human DDAH2 gene was constructed by authors’ laboratory [20] and the adenovirus encoding β-galactosidase gene (Ad5CMVβ-gal) was the product of Biosciences Clontech (Mountain View, CA, USA). The commercial kits of total cholesterol (TC), triglycerides (TG), low density lipoprotein (LDL) and high density lipoprotein (HDL) were purchased from Zhejiang Dongou Bioengineering Co. Ltd (Wenzhou, Zheijiang, P.R.China). The commercial kits for determination of superoxide dismutase (SOD) activity, malondialdehyde (MDA), NO metabolites nitrite/nitrate and protein contents were purchased from Nanjing Jiancheng Bioengineering Institude (Nanjing, Jiangsu, P.R.China). The polyclonal antibody against DDAH1 or DDAH2 was purchased from Abcam (Cambridge, MA, USA), polyclonal antibodies against β-actin and eNOS from Santa Cruz Biotechnology (Santa Cruz, CA, USA), while polyclonal antibody against phosphorylated-IκB (P-IκB) or total-IκB (T-IκB) from Cell Signaling Technology (Danvers, MA, USA). The ELISA kit for measurement of TNF-α was the product of Yanji Biological Technology Co. Ltd (Shanghai, China). The human embryo kidney cell line (HEK 293) was purchased from American Type Culture Collection (ATCC, Manassas, USA). DMEM and fetal bovine serum (FBS) were obtained from Gibco BRL (Gaithersburg, USA) and Sijiqing Biological Co. Ltd (Hangzhou, Zhejiang, P.R.China), respectively.

### 2.2 Preparation of diabetic animal model

All animal experiments were carried out in accordance with the National Institutes of Health guide (NIH Publications No. 8023, revised 1978) and approved by the Laboratory Animal Care and Use Committee of Central South University and Guangzhou Medical University. Adult male Sprague Dawley (SD) rats (220 ± 10g) were provided by the Animal Services of Central South University (Changsha, Hunan, P. R. China). After one week of adaptive feeding, rats were randomly divided into normal control (Control), diabetes (DM), diabetes with PDTC treatment (DM+PDTC) and PDTC control (PDTC) groups. Diabetes was induced by a single intraperitoneal injection of streptozotocin (STZ, 60 mg/kg) to rats as previously described [9]. Onset of diabetes was confirmed by the blood glucose level ≥16 mmol/L of random measurement every other day for 2 consecutive times. An equal volume of citrate buffer was administrated to control rats. After diabetic rat model was established successfully (about 5 days after STZ injection), half of control and diabetic rats were treated with PDTC (10 mg/kg/d) dissolved in drinking water for 8 consecutive weeks, and the dose of PDTC was chosen according to the previous reports of minimum dose exerting antioxidant effect or NF-κB inhibition [21–22]. No rat died during the treatment period because all efforts were made to minimize suffering of animals during the whole experiment. All rats were caged separately with food and water ad libitum under a 12h light /12h dark cycle. Bedding was changed twice a day for cleanliness to minimize the risk of infection since diabetic animals had polyuria. All animals were monitored daily for their well-being and checked on a weekly basis for their body weight and appearance.

### 2.3 Assay of blood biochemistry

At the end of feeding 8 weeks, rats were sacrificed under anesthesia with sodium pentobarbital (30 mg/kg, i.p.). Blood samples were collected from carotid artery of rats and then centrifuged at 3000×g for 15 min at 4°C. Plasma and serum were then separated and stored at -80°C for biochemical measurement.

Plasma glucose concentration was measured within 1h after blood sample collection by glucose oxidase method. Serum lipid profiles including TC, TG, LDL and HDL were assayed spectrophotometrically with respective commercial kits. Serum ADMA level was detected by high-performance liquid chromatography (HPLC) as previously described [23]. Briefly, Serum (0.1 ml) was mixed with 5-sulpfosalicylic acid (2 mg), stored at 4°C for 10 min to precipitate protein and removed by centrifugation at 2500×g for 15 min (4°C). The supernatant was used for the measurement of ADMA.

### 2.4 Measurement of endothelium-dependent and -independent relaxation

After blood was taken, the thoracic aorta was immediately isolated and put in a plate filled with pre-cooled (4°C) Krebs-Henseleit buffer containing NaCl 118.3, KCl 4.7, CaCl_2_ 2.5, MgSO_4_ 1.2, KH_2_PO_4_ 1.2, NaHCO_3_ 25.0, glucose 11.0 (mmol/L) with aeration of 95% O_2_ and 5% CO_2_. After cleaned off the perivascular connective tissue and fat tissue, the thoracic aorta was cut into rings of 3~4 mm in length for vascular function detection and *ex vivo* gene transfection.

The endothelium-dependent relaxation of isolated aortic rings from control and diabetic rats were measured before and after transduction of adenovirus as previously described [23]. The rings were suspended horizontally between two stirrups in organ bath chambers filled with 5 ml Krebs’ solution at 37°C and aerated continuously with 95% O_2_ plus 5% CO_2_. One stirrup was fixed to an anchor and the other was connected to a force transducer. The isometric tension was recorded by the MS-302 biological signal record and analysis system, and the solution in the bath chambers was changed every 15 min. After equilibration for 90 min under 2 g resting tension, the aortic rings were first challenged with 60 mmol/L KCl until a reproducible maximal contractile response was achieved, and then the contraction of aortic rings response to a submaximal concentration of phenylephrine (1μmol/L) was induced following 30 min of equilibration. Subsequently, the endothelium-dependent relaxation responses to cumulative concentrations of acetylcholine (0.03~3 μmol/L) were detected at the plateau phase of phenylephrine contraction. Finally, the endothelium-independent relaxation response to cumulative concentrations of sodium nitroprusside (0.001~3 μmol/L) was also tested in aortic rings after equilibration for another 30 min.

### 2.5 Construction of recombinant adenovirus and *ex vivo* gene transfection

Previously constructed pAdeno-hDDAH2 plasmid was linearized and transfected into HEK 293 cells (15 passages; expressing E1 region of Ad5) through SuperFect^®^ (Hamburg, Germany) for propagation. The primary viral stock was derived from the cell pellets by repeated freezing and thawing cycles. Virus were purified by double cesium chloride (CsCl) step gradient ultracentrifugation and desalted by dialysis. Viral titer was measured by absorbance at 260 nm (vp/ml = OD260±dilution±1.1±10^12^) [24]. The control adenovirus encoding β-galactosidase gene (Ad5CMVβ-gal) was propagated, isolated and quantified as described above. Viral stocks were kept at -80°C for use.

According to the method as previously described [20], isolated aortic rings from diabetic and control rats were incubated with the suspension of Ad5CMV-hDDAH2 (1.5×10^10^ pfu/ml), Ad5CMVβ-gal (1.5×10^10^ pfu/ml) or vehicle (PBS) for 2 h at 37°C, respectively. After rinsing of PBS, aortic rings were kept in DEME media contained 10% fetal calf serum for 24 h under 37°C with continuous aeration of 95% O_2_ and 5% CO_2_. The endothelium-dependent relaxation and DDAH activity were then measured in these aortic rings following the incubation.

### 2.6 Determination of DDAH activity and nitrite/nitrate contents

Vascular DDAH activity was measured based on the method previously established by the authors’ laboratory [12]. Rat aortic tissue was homogenized in ice-cold phosphate buffer (0.1 mol/l, pH 6.5) and then centrifuged by 3500×g for 30 min at 4°C. The supernatant was then assayed for DDAH activity by the L-citrulline formation from ADMA. One unit of the enzyme was defined as the amount that catalyzed formation of 1 μmol/L L-citrulline from ADMA per min at 37°C.

The contents of nitrite/nitrate, the stable end product of NO, in vascular tissue homogenate were detected with a commercial kit as previously described [13]. Nitrate was first converted into nitrite by the aspergillus nitrite reductase, and then the total nitrite was measured with the Griess reagent. The absorbance was determined at 550 nm with a spectrophotometer. The protein content of the supernatant was measured by Coomassie brilliant blue method and used to normalize DDAH activity.

### 2.7 Detection of MDA content and SOD activity

The content of MDA, derived from lipid peroxidation, in aortic homogenates was determined with the commercial kit, and SOD activity was assayed in the homogenates by monitoring the inhibition of the autoxidation of hydroxylamine as previously described. One unit of SOD activity was defined as the amount that inhibited autoxidation of hydroxylamine by 50%. Results of MDA contents and SOD activities were normalized to the protein content of aortic homogenates.

### 2.8 Western blotting and ELISA

Western blotting was performed to examine the expression of DDAH1 or DDAH2, eNOS or iNOS, and P-IκB or total IκB proteins in aortas of rats as described previously[25]. Aortic tissue lysates were prepared by homogenization with RIPA buffer, and equivalent amounts of protein (20–30 μg/well) were loaded on 10% sodium dodecyl sulfate polyacrylamide gel electrophoresis before the proteins were transferred onto a PDVF membrane. After membranes were incubated with 5% skimmed milk at room temperature for 2 h, the polyclonal antibodies against DDAH1 or DDAH2, eNOS, P-IκB or total IκB and β-actin were added to incubate with the membranes over night at 4°C, followed by incubation with second goat anti-rabbit polyclonal antibody at room temperature for 2h, respectively. After washing again, protein bands were detected by the method of chemiluminescence and visualized with a Molecular Imager^®^ ChemiDocTM XRS+ System (Bio-Rad Laboratories, Hercules, CA, USA). Serum TNF-α level was measured by a commercial ELISA kit.

### 2.9 Stastical analysis

The maximum relaxation (Emax) value of aortic rings response to cumulative acetylcholine was calculated respectively and expressed by the percentage of phenylephrine contraction. Linear regression from the log dose-response curves was performed to calculate the half-maximum effective concentration (EC_50_) value for acetylcholine-elicited relaxation of aortic rings. Data are expressed as means ± *SEM*. Statistical analysis was carried out with one-way ANOVA and Newman-Keuls test. *P*<0.05 was considered statistically significant.

## 3. Result

### 3.1 PDTC reduced blood glucose rather than serum lipid profiles in diabetic rats

For the objective to confirm the successful establishment of diabetic rat model, blood glucose concentration and serum lipid profiles were assayed in the current study. As presented in Table 1, blood glucose level was significantly elevated whereas the body weight was declined in diabetic rats compared with that in control group (*P<*0.01 *vs* Control). In addition, diabetic rats also presented abnormal lipids metabolism such as lowered serum HDL level (*P<*0.05 *vs* Control) as well as significantly elevated serum concentrations of TG, TC and LDL (*P<*0.01 *vs* Control). Treatment with PDTC for 8 weeks could reduce blood glucose (*P<*0.01 *vs* DM) and increase body weight (*P*<0.05 *vs* DM) but failed to reverse the disorders of lipid metabolism in diabetic rats (*P>*0.05 *vs* DM). However, PDTC had no significant effect on body weight, blood glucose and serum lipid profiles in control rats (*P>*0.05 *vs* Control).

**Table 1:** Effect of PDTC on blood glucose and serum lipid profiles in diabetic rats.

Groups	Body weight(g)	Glucose(mmol/L)	TC(mmol/L)	TG(mmol/L)	LDL(mmol/L)	HDL(mmol/L)
**Control**	394.60±7.83	8.22±0.85	1.92 ± 0.10	0.63 ± 0.06	1.09 ± 0.10	0.74 ± 0.06
**DM**	210.33±5.81**	28.47±2.44**	4.26 ± 0.26**	1.70 ± 0.17**	2.37 ± 0.38*	0.48 ± 0.05*
**DM+PDTC**	236.80±8.25^#^ **	13.25±1.55^##^ *	3.76 ± 0.29**	1.40 ± 0.12**	1.98 ± 0.37*	0.55 ± 0.05*
**PDTC**	390.00±8.12	8.61±0.88	1.98 ± 0.15	0.66 ± 0.08	1.05 ± 0.13	0.72 ± 0.07

Male Sprague-Dawley rats were randomly divided into normal control, diabetic (DM), pyrrolidine dithiocarbamate (PDTC)-treated DM (DM+PDTC) and PDTC control (PDTC) groups. Diabetic model was induced by a single intraperitoneal injection of streptozotocin(STZ, 60 mg/kg) to rats. Onset of diabetes was confirmed by the blood glucose level ≥16 mmol/L of random measurement every other day for 2 consecutive times. PDTC was administrated in drinking water at the dose of 10 mg/kg/d for 8 weeks. Serum levels of lipids profiles including total cholesterol (TC), triglyceride (TG), low density lipoprotein (LDL) and high density lipoprotein (HDL) were assayed using their commercial kits. Data are expressed as mean ± *SEM*, n = 5;

*P<0.05,

**P<0.01 *vs* Control;

^#^*P*<0.05,

^##^*P*<0.01 *vs* DM.

### 3.2 PDTC improved impaired vasodilation of diabetic rats

**Fig 1:**
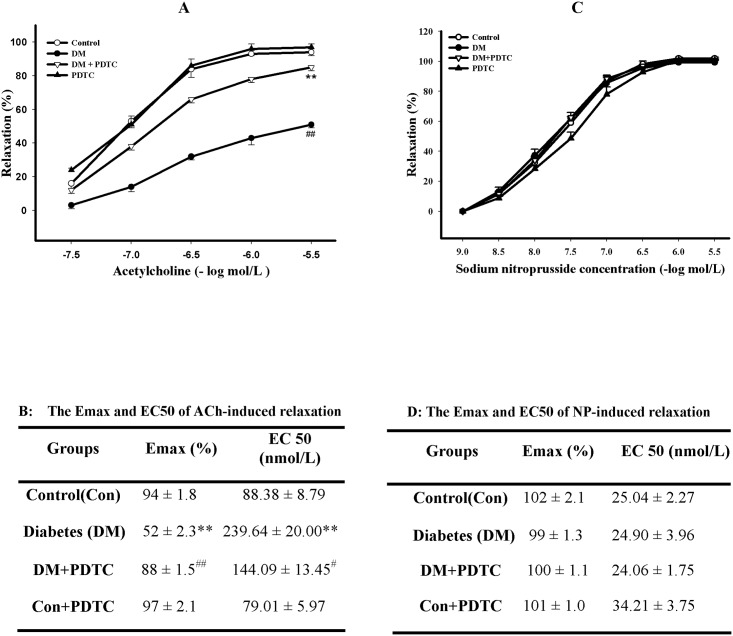
PDTC treatment ameliorated the impairment of endothelium-dependent relaxation in aortic rings from diabetic rats. Endothelium-dependent and -independent relaxation responses to cumulative concentrations of acetylcholine (ACh, Fig 1A) and sodium nitroprusside (SNP, Fig 1C) were measured by recording isometric tension in isolated aortic rings from control, diabetic (DM), pyrrolidine dithiocarbamate (PDTC)-treated diabetic (DM+PDTC) and PDTC-treated control (PDTC) rats. The maximum relaxation (E_max_) and the half-maximum effective concentration (EC_50_) values of ACh-elicited endothelium-dependent relaxation (Fig 1**B**) and SNP-elicited endothelium-independent relaxation (Fig 1**D**) were calculated. Data are expressed as mean ± *SEM*, n = 5~6. *P<0.05, **P<0.01 *vs* Control; ^*#*^*P<*0.05, ^*##*^*P<*0.01 *vs* DM.

To evaluate the effect of PDTC treatment on endothelial dysfunction of diabetic rats, thoracic aortic rings were isolated to assess the relaxation response to cumulative acetylcholine (0.003~3 mol/L) in organ chamber experiment. Although the contraction of aortic rings response to phenylephrine (1μmol/L) was not significantly different among the groups (*P*>0.05, data not shown), the NO-mediated and endothelium-dependent relaxation response to acetylcholine of aortic rings was significantly impaired in diabetic rats compared to control rats, as shown by the lower E_max_ and higher EC_50_ value of acetylcholine-elicited relaxation (*P*<0.01 *vs* Control, Fig 1A & 1B), indicating impairment of endothelium-dependent vasodilation in diabetic rats. After treatment with PDTC for 8 weeks, the impaired endothelium-dependent relaxation of diabetic aorta was remarkably ameliorated, showing as an increase of the E_max_ and decrease of the EC_50_ value (*P*<0.01 *vs* Diabetes, Fig 1A & 1B). However, PDTC failed to change the endothelium-dependent relaxation of control rats (*P>*0.05 *vs* Control, Fig 1A & 1B). PDTC did not affect the endothelium-independent relaxation of aortas responses to cumulative concentrations of sodium nitroprusside (0.001~3 μmol/L), as shown by no difference in both E_max_ and EC_50_ value of sodium nitroprusside-elicited vessel relaxation between groups (*P>*0.05, Fig 1C & 1D).

### 3.3 PDTC normalized the disorder of DDAH/ADMA/NO pathway in diabetic rats

**Fig 2:**
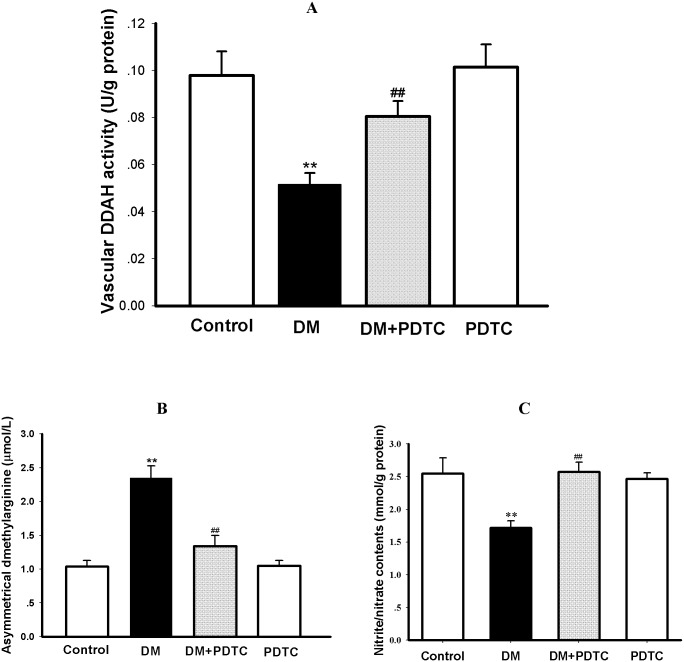
PDTC treatment normalized the disorder of DDAH/ADMA/NO pathway in diabetic rats. The activity of dimethylarginine dimethylaminohydrolase (DDAH, Fig 2A) and contents of nitric oxide (NO) metabolites nitrite/nitrate (Fig 2C) in aortas as well as serum concentration of asymmetric dimethylarginine (ADMA, Fig 2B) were determined in control, Diabetic (DM), pyrrolidine dithiocarbamate (PDTC)-treated diabetic (DM+PDTC) and PDTC-treated control (PDTC) rats. Data are expressed as mean ± S*EM*, n = 5~6; **P<0.01 *vs* Control; ^*##*^*P<*0.01 *vs* DM.

To determine whether normalizing the disorder of DDAH/ADMA/NO pathway is involved in the mechanism underlying PDTC protection against diabetes-induced impaired vasodilation, the serum ADMA concentration and its key metabolic enzyme DDAH activity, protein expression of DDAH and NOS as well as contents of NO metabolites nitrite/nitrate in aortic tissue were measured in the present study. As depicted in Fig 2, DDAH activity of thoracic aortas from diabetic rats were significantly lower than that of control rats (*P*<0.01, Fig 2A). Serum ADMA level of diabetic rats was notably higher compared to control group (*P*<0.01, Fig 2B). The contents of nitrite/nitrate, the stable end metabolites of NO and usually used to reflect NO production, were remarkably reduced in diabetic rats when compared with control rats (*P*<0.01, Fig 2C). Treatment with PDTC for 8 weeks not only successfully restored the vascular DDAH activity of diabetic aortas nearly to the normal level but also remarkably lowered serum ADMA level of diabetic rats and normalized nitrite/nitrate contents of diabetic aortas (*P*<0.01 *vs* DM, Fig 2A~2C). In contrast, PDTC per se didn’t affect the vascular DDAH activity and nitrite/nitrate contents as well as serum ADMA level in control rats (*P>*0.05 *vs* Control, Fig 2A~2C).

**Fig 3:**
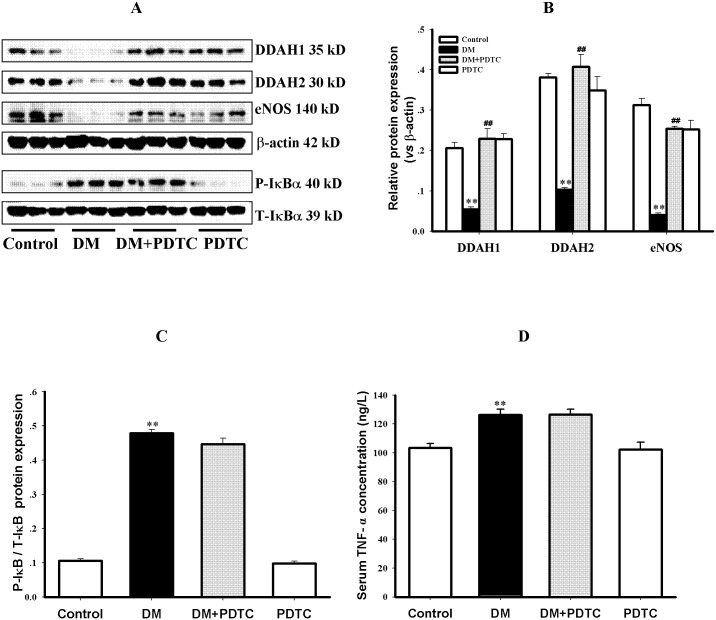
Changes in protein expression of vascular DDAH1, DDAH2, eNOS, P-IκB and serum concentration of TNF-α in diabetic rats. The protein expression of DDAH1, DDAH2, eNOS, P-IκB and T-IκB in thoracic aorta was detected by Western blotting and the serum TNF-α concentration was measured by ELISA. Fig 3A shows the gel electrophoresis bands of indicated protein expression in rat aortas, Fig 3B represents the quantification of relative protein expression *vs* β-actin, and Fig 3C represents the quantification of P-IκB *vs* T-IκB protein expression from 3 independent experiments, respectively. Fig 3D shows the serum TNF-α concentration of rats. Data are expressed as mean ± SEM. n = 3. **P<0.01 *vs* Control. ^##^*P*<0.01 *vs* DM.

The protein expression of DDAH1, DDAH2 and eNOS were decreased in aortic tissues of diabetic rats as compared to control rats (*P<*0.01, Fig 3A & 3B). Treatment with PDTC for 8 weeks enhanced the protein expression of eNOS, DDAH 1 and 2 in aortas of diabetic rats (*P<*0.01, Fig 3A & 3B), but PDTC treatment has no effect on these protein expression in aortas of control rats (*P*>0.05, Fig 3A & 3B).

### 3.4 PDTC didn’t affect NF-κB activation and inflammatory response in diabetic rats

To determine whether anti-inflammatory action is implicated in the mechanism underlying PDTC protection against diabetes-induced impaired vasodilation, the phosphorylation of IκB in aortas and serum TNF-α concentration were detected to reflect the activation of NF-κB and inflammatory response in diabetic rats. As shown in Fig 3A, 3C and 3D, the protein expression of P-IκB was upregulated in aortas (*P*<0.01), and the level of TNF-α in serum was significantly higher in diabetic rats than that in control rats (*P*<0.01), indicating the activation of vascular NF-κB and an inflammatory response in diabetic rats. However, treatment with PDTC at the dosage of 10 mg/kg/d for 8 weeks neither reduced the protein expression of P-IκB in aortas nor lowered TNF-α levels in serum of diabetic rats (*P*>0.05 *vs* DM), indicating that anti-inflammatory action was not implicated in the mechanism of PDTC protection against impaired vasodilatation of diabetic rats in the current study. PDTC per se didn’t affect the phosphorylation of IκB and serum TNF-α concentration in control rats (*P>*0.05 *vs* Control, Fig 3A, 3C and 3D).

### 3.5 PDTC alleviated oxidative stress in aortas of diabetic rats

**Fig 4:**
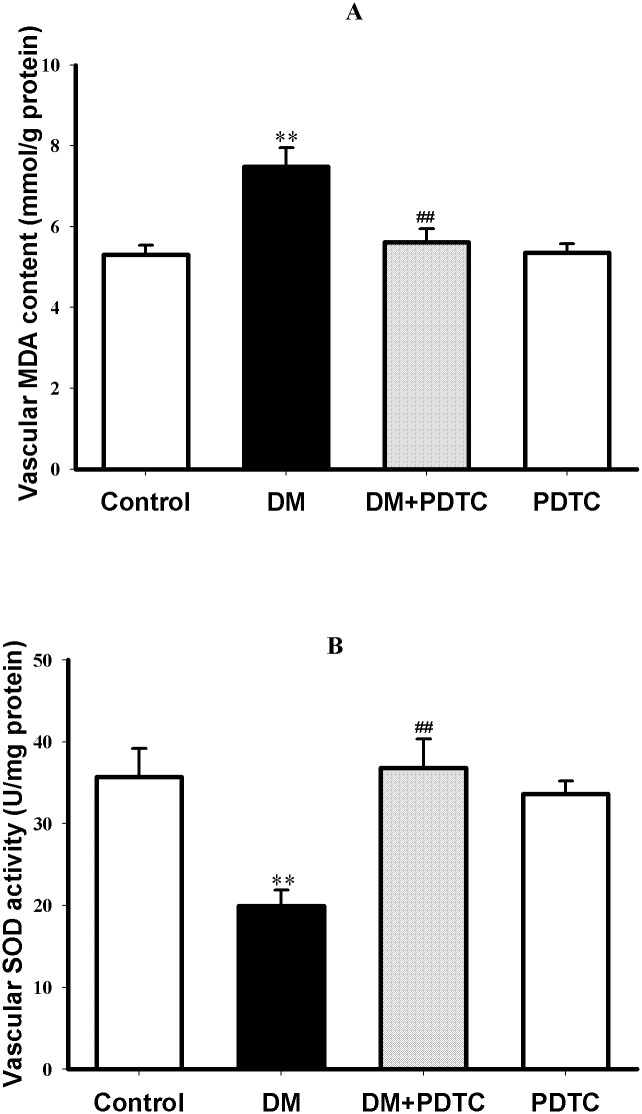
PDTC treatment alleviated oxidative stress in aortas of diabetic rats. Oxidative stress was reflected by the increased content of the lipid peroxidation product malondialdehyde (MDA, Fig 4A) and the decreased activity of antioxidant enzyme superoxide dismutase (SOD, Fig 4B) in aortic tissue of rats. Data are expressed as mean ± SEM. n = 6. **P<0.01 *vs* Control. ^##^*P*<0.01 *vs* DM.

To evaluate whether an antioxidant effect is involved in the mechanism of PDTC protection against diabetes-induced impairment of vasodilation, both the content of lipid peroxidation product MDA and activity of antioxidant enzyme SOD were measured to reflect oxidative stress. In comparison with control rats, a higher MDA content and lower SOD activity were observed in the aortic tissues of diabetic rats (*P*<0.01, Fig 4A & 4B), indicating an enhancement of oxidative stress in diabetic aortas. Treatment with PDTC for 8 weeks not only decreased vascular MDA content but also increased aortic SOD activity in diabetic rats (*P*<0.01 *vs* DM, Fig 4A & 4B), indicating that the alleviation of oxidative stress was involved in the protection of PDTC against diabetes-induced impairment of vasodilatation. However, PDTC per se did not affect the oxidative stress in aortas of control rats (*P*<0.01 *vs* DM, Fig 4A & 4B).

### 3.6 *Ex vivo* DDAH2 gene transfection reversed the inhibition of vascular DDAH2 activity and impairment of vasodilation in diabetic rats

**Fig 5:**
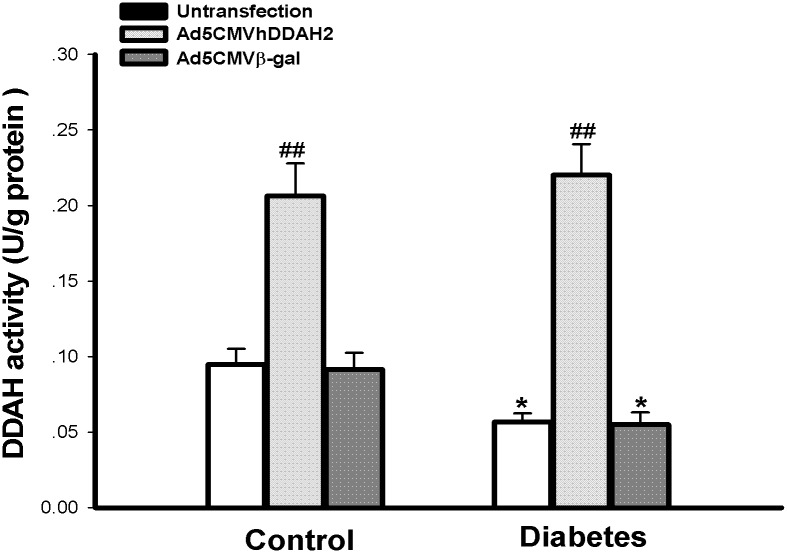
*Ex vivo* DDAH2 gene transfection increased DDAH activity in isolated aortas from diabetic and control rats. Aortic rings were incubated with 1.5 × 10^10^ pfu/ml of Ad5CMVDDAH2, Ad5CMVβ-gal or PBS (Untransfection) for 2 h at 37°C and then kept in DMEM medium for 24h, respectively. The activity of DDAH was determined by the conversion of asymmetric dimethylarginine to L-citrulline with aortic tissue homogenates. Data are expressed as mean ± *SEM*, n = 5; *P<0.05 *vs* respective control; ^##^*P*<0.01 *vs* group-matched untransfection or Ad5CMVβ-gal transfection.

To compare the protective effect of PDTC on diabetes-induced impairment of vasodilation with that of vascular DDAH overexpression, *ex vivo* DDAH2 gene transferring to isolated aortic rings of diabetic and control rats was performed in this study. As shown in Fig 5, *ex vivo* DDAH2 gene transfection remarkably increased the vascular DDAH activity in both control and diabetic rats (*P*<0.01 *vs* Untransfection or β-gal transfection), whereas *ex vivo* β-gal gene transfection had no significant effect on vascular DDAH activity either in diabetic or control groups (*P*>0.05 *vs* Untransfection).

**Fig 6:**
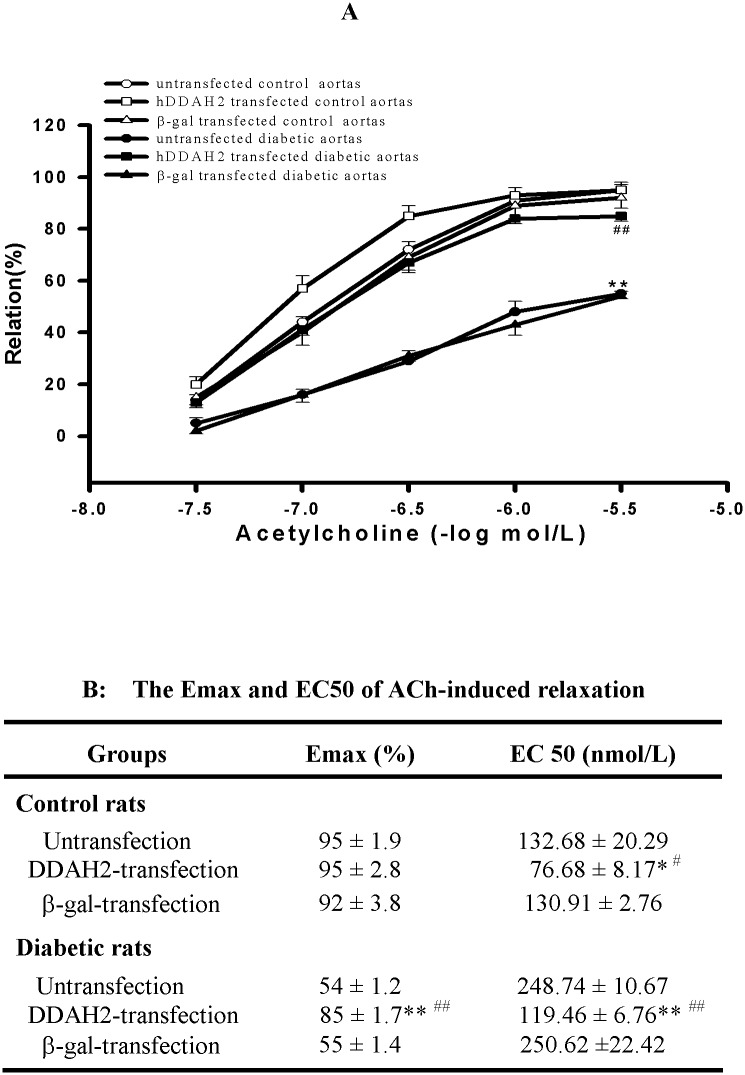
*Ex vivo* human DDAH2 gene transfection normalized the impairment of endothelium-dependent relaxation in aortic rings from diabetic rats. Aortic rings were incubated with 1.5 × 10^10^ pfu/ml of Ad5CMVDDAH2, Ad5CMVβ-gal or PBS (Untransfection) for 2 h and then kept in DMEM medium for 24h at 37°C, respectively. Endothelium-dependent relaxation responses to cumulative concentrations of acetylcholine (ACh) were measured by recording isometric tension in isolated aortic rings before (Untransfection) and after (DDAH2 or β-Gal) gene transfection (Fig 6**A**). The E_max_ and EC_50_ values of ACh-elicited endothelium-dependent relaxation were calculated (Fig 6**B**). Data are expressed as mean ± *SEM*, n = 5. *P<0.05, **P<0.01 *vs* respective control; ^#^*P*<0.05, ^##^*P*<0.01 *vs* group-matched untransfection or Ad5CMVβ-gal transfection.

Consistent with vascular DDAH activity, *ex vivo* DDAH2 gene transferring to diabetic aortic rings almost normalized the impairment of endothelium-dependent relaxation, presenting as an increased E_max_ of vascular relaxation (*P*<0.01 *vs* Untrasfected aortic rings of Diabetic group, Fig 6A) and decreased EC_50_ of acetylcholine (*P*<0.01 *vs* Untrasfected aortic rings of Diabetic group, Fig 6B). While *ex vivo* DDAH2 gene transferring to control aortic rings had no significant effect on the E_max_ of vascular relaxation (*P*>0.05 *vs* Untrasfected aortic rings of Control group, Fig 6A), but it lowered the EC_50_ of acetylcholine (*P*<0.05 *vs* Untrasfected aortic rings of Control group, Fig 6B). However, *ex vivo* β-gal gene transferring didn’t change the endothelium-dependent relaxation of aortic rings from diabetic or control rats (*P*>0.05 *vs* Untransfection). These results indicated that the *ex vivo* DDAH2 gene transfection could reverse endothelium dysfunction in thoracic aortas of diabetic rats, and its therapeutic effect was similar to that of PDTC treatment.

## 4 Discussion

The present study reveals for the first time that PDTC could protect against the impairment of endothelium-dependent vasodilatation in diabetic rats, and this protection was associated with the preservation of vascular DDAH activity as well as the decrease of endogenous ADMA accumulation and increase of NO content. Furthermore, this protection of PDTC was very similar to the effect of *ex vivo* DDAH2 gene transfection on the impaired vasodilation of diabetic rats.

Diabetes mellitus is characterized by disorders of glucose and lipid metabolism. Elevated blood glucose level and disordered serum lipid profiles were observed in diabetic rats, indicating that diabetic model was successfully induced by STZ injection in the present study. Treatment with PDTC for 8 weeks could reduce the blood glucose level in diabetic rats. This result is consistent with the previous report by Stosic-Grujicic *et*. *al*.[26]. It has been well documented that PDTC is a NF-κB inhibitor and imposes antioxidative effect as a sulfydryl-containing compound [15–17]. Recent studies have also demonstrated that PDTC could protect pancreatic β-cell against apoptosis induced by oxidative injury or inflammatory cytokines in various diabetic animal models by its inhibition of NF-κB activation [27] and antioxidative effects [28], resulting in the increase of pancreatic β-cell mass and function. According to these studies, it can be speculated that underlying mechanism for PDTC lowering blood glucose level of diabetic rats in the current study may be related to the protection of pancreatic β-cell against STZ injury by its antioxidant or anti-inflammatory properties.

Endothelial dysfunction is an early manifestation of diabetes and plays a pivotal role in the development of diabetic cardiovascular complications. It is, hence, of importance to look for medicines of protection against diabetic endothelial dysfunction for the prevention of diabetic cardiovascular complications. In consistent with a larger number of previous studies [7–10], the present study proved that the endothelium-dependent relaxation of thoracic aortas response to acetylcholine was significantly impaired in diabetic rats compared to control rats. More strikingly, this study revealed that PDTC treatment could remarkably ameliorate the impairment of endothelium-dependent relaxation in diabetic rats, evidenced by enhances of the maximum relaxation (with a higher E_max_) and sensitivity (with a lower EC_50_) of thoracic aortas response to acetylcholine. However, PDTC treatment neither affected endothelium-independent relaxation of aortas responses to cumulative concentrations of sodium nitroprusside nor changed contraction of aortas response to phenylephrine in both diabetic and control rats. These results indicate that PDTC protection of vascular relaxation against diabetic injury is limited to the endothelium. Previous study has reported that treatment with PDTC could inhibit expression of endothelial adhesive molecules and adhesion of monocytes induced by high glucose in cultured human umbilical vein endothelial cells [29] as well as reduce the ROS generation and improve mitochondrial function in cardiomyocytes of db/db mice [30]. Furthermore, PDTC treatment at the dosage of 30 mg/kg/d for 1 week has also been reported to reduce prostanoid-induced vascular contraction by lowering synthesis and release of endothelium-derived contracting factor in mesenteric arteries of aged type 2 diabetic rats [31]. To the best of our knowledge, there has been no study to show PDTC protection against the impairment of endothelium-dependent relaxation in diabetes except this study. Our findings suggest that the amelioration of PDTC on the impairment of endothelium-dependent vasorelaxation response to acetylcholine in diabetic rats is through enhancement of endothelium-derived NO bioavailability since acetylcholine-elicited vasorelaxation is endothelium-dependent and NO-mediated. Furthermore, the present study also provides the evidence that PDTC treatment increased the contents of NO metabolites nitrite/nitrate, reflecting the enhancement of NO content in aortic tissues of diabetic rats. However, the underlying mechanism by which PDTC increases NO bioavailability and improves diabetes-induced impaired vasodilation remains unknown.

The increase of NO bioavailability in endothelial cells mainly includes two aspects: one is to reduce the oxidative inactivation of NO, and another is to enhance the synthesis of NO in endothelial cells. It has been demonstrated that oxidative inactivation of NO was responsible for the endothelial dysfunction in spontaneously hypertensive stroke-prone rats [32], and reduced NO synthesis by eNOS accounted for the endothelial dysfunction in diabetic patients and animal models [33]. ADMA has been recognized as an endogenous inhibitor of NOS and independent risk factor for endothelial dysfunction [5]. Since DDAH is the key enzyme of endogenous ADMA degradation, inhibition of vascular DDAH activity has been reported to be involved in diabetic endothelial dysfunction [10,34], and DDAH1 or DDAH2 knockout mice showed endothelial dysfunction or endogenous ADMA accumulation [35–36]. Thus, the present study investigated the effect of PDTC on serum endogenous ADMA concentration and vascular DDAH activity as well as protein expression of DDAH and eNOS in diabetic rats to explore the mechanisms by which PDTC increases NO bioavailability resulting in the improvement of diabetes-induced impaired vasodilation. Results showed that PDTC treatment not only significantly attenuated the decreases of vascular DDAH activity, DDAH and eNOS protein expression but also lowered serum ADMA concentration in diabetic rats. Others have reported that PDTC could prevent the inhibition of DDAH activity and accumulation of endogenous ADMA in cultured endothelial cells induced by homocysteine [19] and oxidized LDL [18]. Taking together, our findings indicate that preservation of DDAH activity or protein expression and consequent reduction of endogenous ADMA accumulation contribute to the mechanisms by which PDTC increases NO bioavailability and improves impaired vasodilation of diabetic rats.

To verify that preservation of vascular DDAH activity and its protein expression being involved in the mechanism underlying PDTC protection against diabetes-induced impaired vasodilation, *ex vivo* DDAH2 gene transferring to isolated aortic rings of diabetic rats was performed to compare its effect with that of PDTC treatment on the diabetes-induced impaired vasodilation in the current study. As expected, it was found that the protective effect of PDTC against diabetes-induced impairment of vasodilation was very similar to that of *ex vivo* DDAH2 gene transfection, which increased vascular DDAH activity and improved the impairment of endothelium-dependant relaxation in diabetic rat aortas. I*n vivo* DDAH2 gene transfection has been demonstrated to reduce plasma ADMA concentration and ameliorate ADMA-induced functional and histological injury in coronary microvessels of transgenic mice [37]. These results support our opinion of that DDAH2 plays a critical role in the regulation of endogenous ADMA accumulation and endothelial function. The analogical investigation in the present study suggests that PDTC treatment possesses similar effects or mechanisms to DDAH2 gene transfection underlying their amelioration of diabetes-induced impaired vasodilation.

As for the further mechanism by which PDTC preserves vascular DDAH activity or protein expression remains uncertain. But it may be speculated to be associated with its antioxidant property because PDTC is a sulfydryl-containing compound and has been proved to possess the antioxidant and free radical-scavenging activities [17]. Previous study had demonstrated that PDTC could prevent the oxidative stress and endoplasmic reticulum stress induced by hydrogen peroxide or ADMA in rat hepatoma cells [38]. This study also proved that PDTC treatment could alleviate oxidative stress, reflected by the decrease of lipid peroxidation product MDA content and increase of antioxidant enzyme SOD activity, in aortas of diabetic rats. More importantly, DDAH activity and expression have been proved to be redox-regulated [39]. Several reports have highlighted the protective effects of antioxidants on DDAH activity or expression in cultured endothelial cells incubated with high glucose [10], isolated rat aortas treated with glycated bovine serum albumin [11] and spontaneously hypertensive rats [40]. Taken together, antioxidant effect may account for the mechanism under which PDTC preserved vascular DDAH activity or expression in aortas of diabetic rats.

Although it has been reported that PDTC inhibits NF-κB activation which is involved in β-cell dysfunction or mitochondrial dysfunction [27,30], it is of significance to determine whether inhibition of NF-κB activation is implicated in the mechanism underlying PDTC preserving vascular DDAH activity in diabetic rats. The phosphorylation of IκB in aortas and serum TNF-α concentration were detected to reflect the activation of NF-κB and inflammatory response in the current study. The increases of both vascular IκB phosphorylation and serum TNF-α concentration were observed in diabetic rats, indicating that there are the activation of vascular NF-κB and an inflammatory response in diabetic rats. However, PDTC treatment at the dosage of 10 mg/kg/d neither reduced the phosphorylation of IκB protein in aortas nor lowered the concentration of TNF-α in serum of diabetic rats. These results indicate that the anti-inflammatory effect was not involved in the mechanism under which PDTC preserved vascular DDAH activity or protein expression in diabetic rats. Similar results had been reported in the tumor tissue of Tumor-Bearing Mice and in nephric tissue of polycystic kidney rats [41–42]. The reason may be due to the dosage of PDTC too small and further research should be conducted to confirm this point in future.

In summary, the present study reveals that treatment with PDTC for 8 weeks ameliorates diabetes-induced impairment of endothelium-dependent vasodilatation in STZ-induced diabetic rats. The underlying mechanisms might be related to preservation of vascular DDAH activity resulting in the reduction of endogenous ADMA and increase of NO bioavailability in endothelium through its antioxidant action. These results provide the new insight into the understanding of mechanisms underlying PDTC protection against diabetes-induced impairment of endothelium-dependent vasodilation. This new insight may lead to the discovery of vascular DDAH activity as potential therapeutic targets for the treatment of diabetic cardiovascular complications and development of some new medicines like PDTC for treatment of diabetes-induced impairment of endothelium-dependent vasodilatation and prevention of diabetic cardiovascular complications.

## Supporting information

S1 Data Minimal data. (XLS)
